# Mini‐Open Biceps Tenodesis Using an Onlay Technique With Enthesis Growth Augment

**DOI:** 10.1002/atn2.70167

**Published:** 2026-07-14

**Authors:** Micah Thatcher, Jack Carfagno, Steven Fisher, John Pignataro, Ruchir Nanavati, Christopher Difiore, Sean McMillan

**Affiliations:** ^1^ Jefferson Health, New Jersey Stratford New Jersey U.S.A.; ^2^ Inspira Health Vineland New Jersey U.S.A.; ^3^ Virtua Health Marlton New Jersey U.S.A.

## Abstract

Acute ruptures of the long head of the biceps tendon are a frequent cause of anterior shoulder pain, weakness, and cosmetic deformity. Onlay constructs for long head of the biceps tendon tenodesis with suture anchors or buttons have shown comparable clinical outcomes to traditional inlay techniques. Complication rates with the onlay technique are generally low and are thought to result from failure of enthesis healing. This technique describes a biologically augmented onlay biceps tenodesis using a demineralized bone matrix enthesis implant (EnFix, Tetrous, Sherman Oaks, CA). The demineralized bone matrix implant provides osteoconductive and osteoinductive properties to enhance tendon‐to‐bone healing by promoting enthesis regeneration. This method offers a reproducible and biologically enhanced alternative for proximal biceps tenodesis that may optimize enthesis healing and reduce the risk of failure.

## Graphical abstract

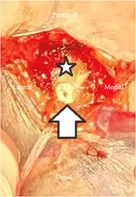

**VIDEO 1:** Video description of the surgical technique employed to perform a biceps tenodesis with enthesis augment in supine position. Through a 5‐cm anterior incision at the level of the pectoralis major tendon, the proximal long head of the biceps tendon is identified, delivered, and whipstitched. A unicortical socket is drilled for placement of a demineralized bone matrix enthesis augment, after which the tendon is secured with a suture button 1 cm proximal to the augment and fixation is confirmed through full elbow range of motion. Video content can be viewed at https://doi.org/10.1002/atn2.70167.

Acute ruptures of the long head of the biceps tendon (LHBT) are a frequent source of anterior shoulder pain and functional impairment. Although nonoperative management can be considered in low‐demand patients, surgical management aims to alleviate pain and minimize cosmetic and functional deficits.^1^, ^2^, ^3^ The onlay method of tenodesis uses suture anchors or button implants to secure the tendon directly onto the cortical surface of the humerus. A systematic review indicates that onlay techniques yield improvements in patient‐reported outcome measures, pain relief, and return to sport/activity rates.^4^

The EnFix (Tetrous, Sherman Oaks, CA) implant is composed of demineralized bone matrix (DBM), possessing osteoconductive and osteoinductive properties.^5^ DBM has shown efficacy in increasing tendon‐to‐bone healing rates in rotator cuff repairs, with improved structural integrity and reduced failure rates.^5^, ^6^, ^7^ Although its use in proximal biceps tendon tenodesis is still emerging, DBM implant offers the potential to enhance enthesis formation and biomechanical strength at the tenodesis site, particularly in onlay constructs.

## SURGICAL TECHNIQUE

Following induction of anesthesia, the patient is positioned in the supine position with the operative arm on a hand table. The operative upper extremity is prepped and draped, ensuring full intraoperative mobility of the arm.

A 5‐cm longitudinal incision is made over the anterior aspect of the arm at the level of the pectoralis major tendon. Electrocautery is employed for hemostasis and to carry the dissection down to bone. The proximal LHBT is identified, an Allis clamp is used to gently deliver it through the incision, and a whipstitch is placed to secure it proximally.

**FIGURE 1:**
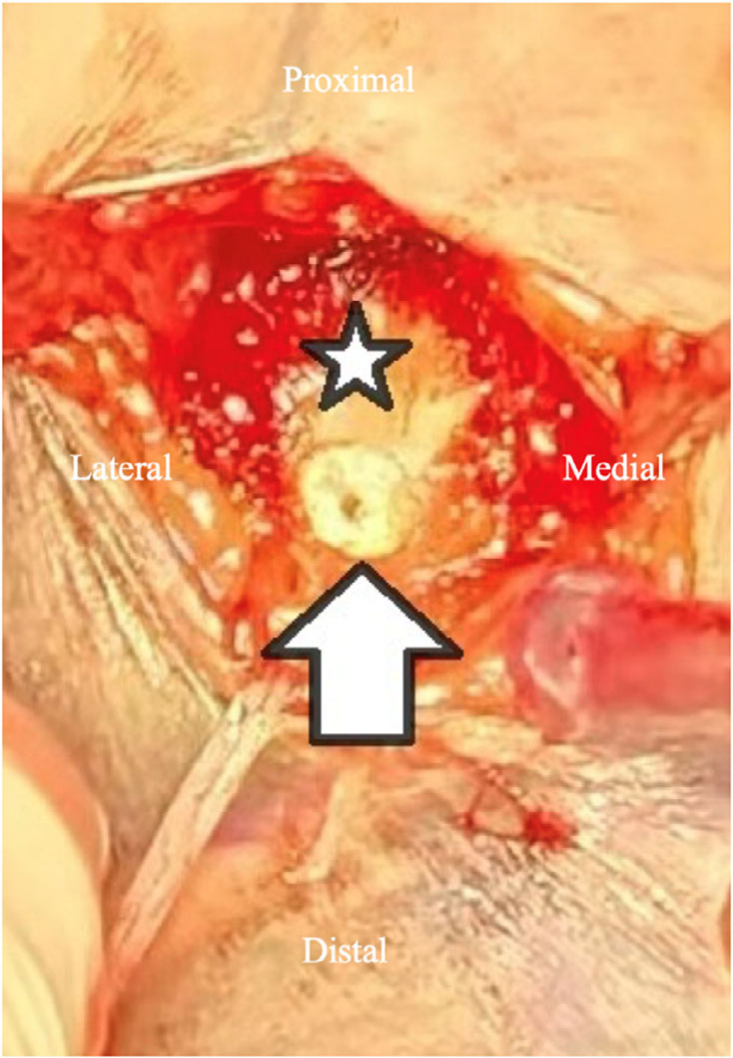
Viewing a right proximal humerus with the head at the top of the image, the enthesis has been seated within the cortical bone flush (arrow) approximately 1 cm inferior to the planned biceps tenodesis site (star).

Traction on the LHBT combined with elbow range of motion is then used to determine the isometric point for the site of tenodesis. The location for DBM implant insertion is one centimeter distal to the site of planned tenodesis (Figure 1). This site is marked and a unicortical guide pin is inserted at the planned enthesis site. The near cortex is reamed with a 4.5 mm cannulated reamer. A 4.5 mm standard awl, followed by a 4.5 mm cutting awl, is then used to create a recessed cavity for implant placement (Table 1).

**TABLE 1:** The Following Table Identifies “Pearls” and “Pitfalls” Associated With Performing an Open Biceps Tenodesis With DBM Enthesis Augmentation

Pearl	Pitfall
Use a 4.5 mm reamer over the placed pin rather than using the punch/awl alone to avoid iatrogenic fracture	Careful spacing of the DBM enthesis from the location of tenodesis anchor placement is important to avoid convergence, recommend 1 cm
Ensure the implant will seat flush with the cortical bone by utilizing the cutting awl after the reamer	Care should be taken to avoid over‐tensioning on the tendon at the tenodesis site to avoid iatrogenic pain or failure
Mark the isometric tensioning point of the planned tenodesis site prior to placing the enthesis	Avoid aggressive early rehabilitation to avoid stressing the repair and enthesis sites

DBM, demineralized bone matrix.

**FIGURE 2:**
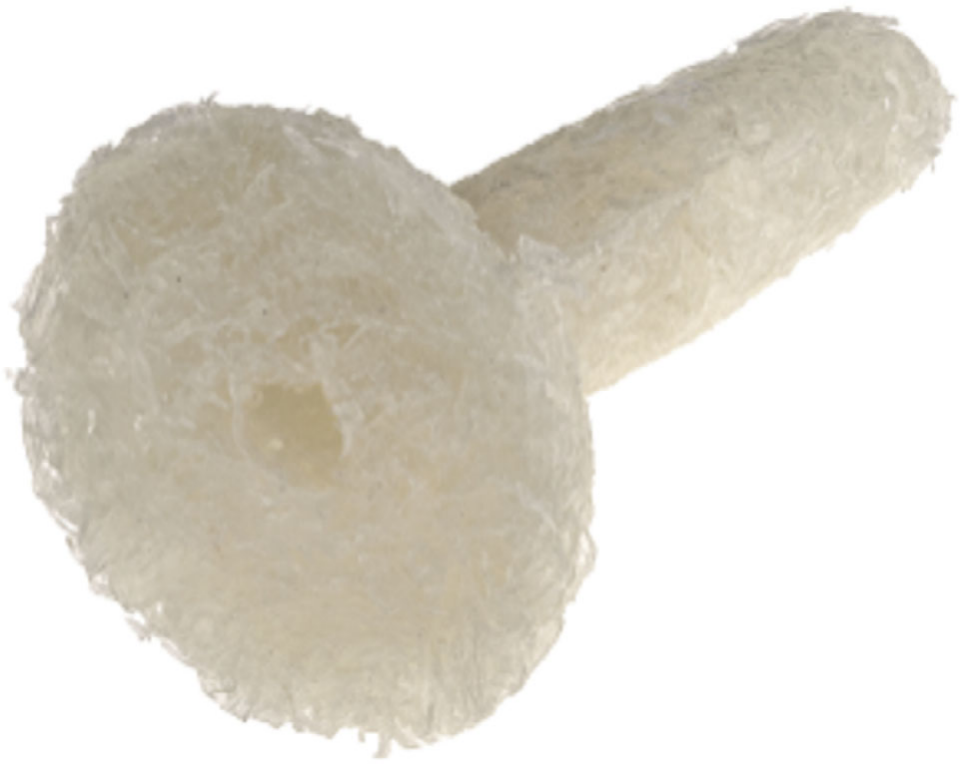
Pictured is the EnFix TAC‐O DMX enthesis. At the widest surface segment it measures 8.5 mm wide with a length of 10 mm. Reprinted with permission from Tetrous.

The enthesis augment, measuring 8.5 mm wide by 10 mm in depth (Figure 2), is positioned on its delivery guide and inserted into the prepared site. An Adson forceps may be used to maintain the augment's position while the inserter is removed.

**FIGURE 3:**
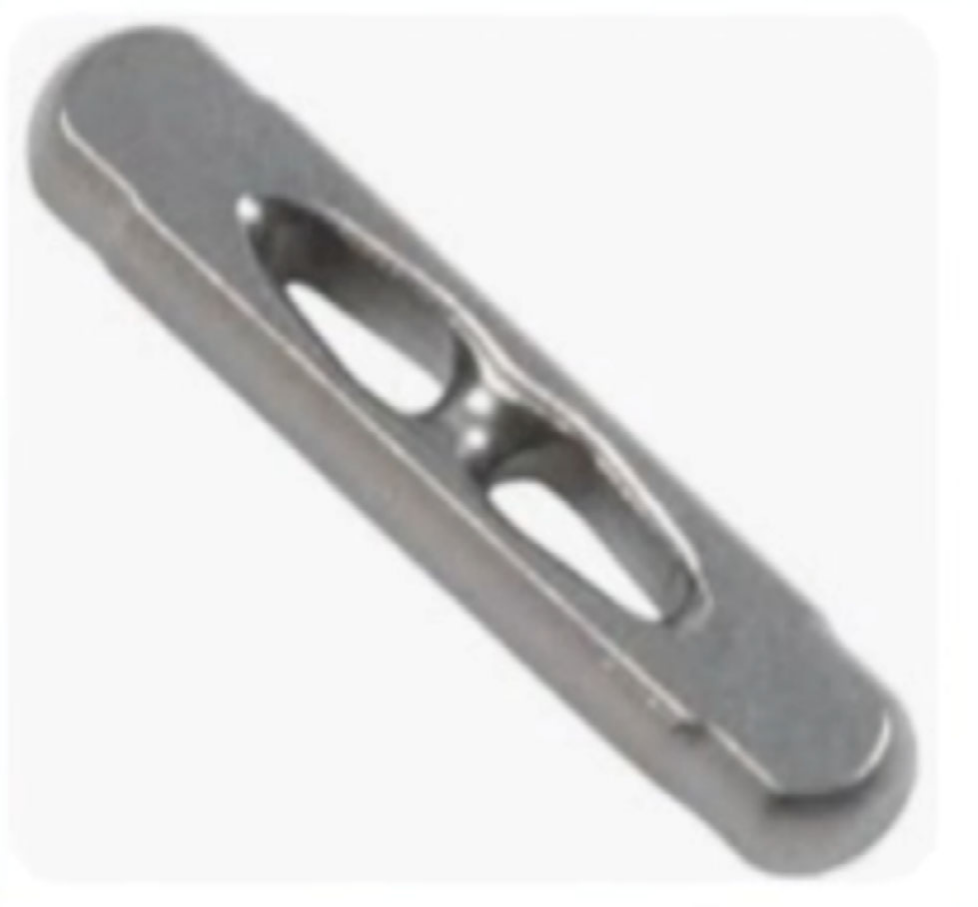
Pictured is an example of a suture button that could be used to secure the biceps tendon to the anterior humeral cortex.

The isometric point for biceps tenodesis is again confirmed and a unicortical hole is drilled at the predetermined site, 1 cm above the placed enthesis (Figure 1). The whipstitched LHBT sutures are passed through the suture button which is then deployed unicortically (Figure 3). The suture limbs are tensioned to lay the proximal LHBT on top of both the enthesis augment and the anterior humeral cortex. The elbow is then taken through a full arc of flexion and extension to confirm both stable fixation of the tenodesis and the absence of restricted range of motion due to excessive or inadequate tensioning (Video 1).

## DISCUSSION

Acute rupture of the LHBT typically presents with sudden anterior shoulder pain, weakness, and a characteristic “Popeye” deformity. Patients may also report cramping, decreased supination strength, and functional limitations, especially in activities requiring elbow flexion or forearm rotation. The onlay technique, which secures the tendon to the cortical surface using suture anchors or button implants, has emerged as a reliable alternative to inlay fixation.^4^

Complication rates with the onlay technique are low with the incidence of persistent Popeye deformity ranging from 0% to 6.7%, and revision rates are similarly low.^8^ Mechanical failure, including anchor pullout or tendon migration, remains rare but is recognized as a potential complication, particularly with all‐suture anchor constructs; in one series, 5.9% of patients required revision for anchor failure.^9^ Persistent anterior shoulder pain, cramping, and subjective weakness are reported in a minority of cases.^8^, ^10^

Failure of enthesis healing during biceps tenodesis using the onlay technique is a common cause of these complications and is primarily due to the inability to biologically recreate the native tendon‐bone interface.^10^ Instead of regenerating the original graded fibrocartilaginous enthesis, the repair site typically heals by forming disorganized scar tissue, which is mechanically inferior and predisposes to retear.^11^ This limitation arises because current surgical techniques and available grafts lack the necessary biological cues to guide proper tissue differentiation and integration at the bone‐tendon interface.^12^

Although the application of the DBM implant in proximal biceps tenodesis is still in its early stages, it shows promise for enhancing enthesis development (Table 2). Preliminary evidence from rotator cuff repair studies suggests that the implant may improve tendon healing and increase construct durability.^5^, ^6^, ^7^ Further clinical research is needed to clarify its ideal indications and performance in the setting of LHBT tenodesis.

**TABLE 2:** Advantages and Disadvantages of Technique

Advantages
The DBM enthesis augment provides osteoconductive and osteoinductive properties that may promote native enthesis formation
This technique builds upon the onlay technique for proximal biceps tenodesis with minimal additional steps and therefore can be easily incorporated into a surgeon's technique

Disadvantages
An additional perforation of the anterior humeral cortex for placement of the enthesis augment carries a theoretical increased risk in peri‐implant fracture
Use of additional implants increases overall cost of surgical procedure

DBM, demineralized bone matrix.

## DECLARATION OF GENERATIVE AI AND AI‐ASSISTED TECHNOLOGIES IN THE WRITING PROCESS

During the preparation of this work the authors used OpenEvidence and ChatGPT for literature and editorial review. After using this tool/service, the authors reviewed and edited the content as needed and take full responsibility for the content of the published article.

## DISCLOSURES

The authors (M.T., J.C., S.F., J.P., R.N., C.D., S.M.) declare that they have no known competing financial interests or personal relationships that could have appeared to influence the work reported in this article.

## References

[1] Cuéllar A , Cuéllar A , Cuéllar R . Editorial commentary: Shoulder biceps tenodesis versus tenotomy: Both show good results and have different indications. Arthroscopy. 2022;38:1843‐1845. 10.1016/j.arthro.2022.01.014 35660180

[2] Anil U , Hurley ET , Kingery MT , et al. Surgical treatment for long head of the biceps tendinopathy: A network meta‐analysis. J Shoulder Elbow Surg. 2020;29:1289‐1295. 10.1016/j.jse.2019.10.021 32037231

[3] Zhang C , Yang G , Li T , et al. Biceps tenodesis better improves the shoulder function compared with tenotomy for long head of the biceps tendon lesions: A meta‐analysis of randomised controlled trials. J Clin Med. 2023;12:1754. 10.3390/jcm12051754 PMC10003204 36902540

[4] Sachs JP , Franzia CH , Mufti YN , et al. Comparable and improved clinical outcomes, pain relief, return to sport, and low Popeye deformity rates in inlay versus onlay open subpectoral biceps tenodesis: A systematic review. Arthroscopy. 2025;41:4278‐4291. 10.1016/j.arthro.2025.03.064 40209831

[5] Villarreal‐Espinosa JB , Saad‐Berreta R , Danilkowicz R , et al. Arthroscopic transosseous‐equivalent double‐row rotator cuff repair augmentation with interpositional demineralized bone fiber implant. Arthrosc Tech. 2024;13:e103133. 10.1016/j.eats.2024.103133 PMC11704921 39780897

[6] He SK , Ning LJ , Yao X , et al. Hierarchically demineralized cortical bone combined with stem cell‐derived extracellular matrix for regeneration of the tendon‐bone interface. Am J Sports Med. 2021;49:1323‐1332. 10.1177/0363546521994511 33667131

[7] Lee WY , Kim YM , Hwang DS , et al. Does demineralized bone matrix enhance tendon‐to‐bone healing after rotator cuff repair in a rabbit model? Clin Orthop Surg. 2021;13:216‐224. 10.4055/cios20099 PMC8173240 34094012

[8] McCrum CL , Alluri RK , Batech M , Mirzayan R . Complications of biceps tenodesis based on location, fixation, and indication: A review of 1526 shoulders. J Shoulder Elbow Surg. 2019;28:461‐469. 10.1016/j.jse.2018.09.005 30573431

[9] Degenhardt H , Pogorzelski J , Themessl A , et al. Reliable clinical and sonographic outcomes of subpectoral biceps tenodesis using an all‐suture anchor onlay technique. Arthroscopy. 2022;38:729‐734. 10.1016/j.arthro.2021.08.033 34508820

[10] Hsu KL , Su WR . Editorial commentary: Failure following biceps long head tenodesis includes Popeye sign, cramping, and tendon migration. Arthroscopy. 2025;41:1314‐1315. 10.1016/j.arthro.2024.09.014 39307326

[11] Jensen PT , Lambertsen KL , Frich LH . Assembly, maturation, and degradation of the supraspinatus enthesis. J Shoulder Elbow Surg. 2018;27:739‐750. 10.1016/j.jse.2017.10.030 29329904

[12] Durtschi MS , Kim S , Li J , et al. Optimizing tissue engineering for clinical relevance in rotator cuff repair. Tissue Eng Part B Rev. 2024;30:559‐569. 10.1089/ten.teb.2023.0320 PMC12947802 38411502

